# Genome-wide age-related changes in DNA methylation and gene expression in human PBMCs

**DOI:** 10.1007/s11357-014-9648-x

**Published:** 2014-05-02

**Authors:** Wilma T. Steegenga, Mark V. Boekschoten, Carolien Lute, Guido J. Hooiveld, Philip J. de Groot, Tiffany J. Morris, Andrew E. Teschendorff, Lee M. Butcher, Stephan Beck, Michael Müller

**Affiliations:** 1 Division of Human Nutrition, Wageningen University, Bomenweg 2, Wageningen, 6703 HD The Netherlands; 2 Medical Genomics, Paul O’Gorman Building, UCL Cancer Institute, University College London, 72 Huntley Street, London, WC1E 6BT UK; 3 Statistical Cancer Genomics, Paul O’Gorman Building, UCL Cancer Institute, University College London, 72 Huntley Street, London, WC1E 6BT UK

**Keywords:** Molecular aging, Epigenetics, DNA methylation, Gene expression, PBMCs, Epigenetic biomarkers of aging

## Abstract

Aging is a progressive process that results in the accumulation of intra- and extracellular alterations that in turn contribute to a reduction in health. Age-related changes in DNA methylation have been reported before and may be responsible for aging-induced changes in gene expression, although a causal relationship has yet to be shown. Using genome-wide assays, we analyzed age-induced changes in DNA methylation and their effect on gene expression with and without transient induction with the synthetic transcription modulating agent WY14,643. To demonstrate feasibility of the approach, we isolated peripheral blood mononucleated cells (PBMCs) from five young and five old healthy male volunteers and cultured them with or without WY14,643. Infinium 450K BeadChip and Affymetrix Human Gene 1.1 ST expression array analysis revealed significant differential methylation of at least 5 % (ΔYO > 5 %) at 10,625 CpG sites between young and old subjects, but only a subset of the associated genes were also differentially expressed. Age-related differential methylation of previously reported epigenetic biomarkers of aging including ELOVL2, FHL2, PENK, and KLF14 was confirmed in our study, but these genes did not display an age-related change in gene expression in PBMCs. Bioinformatic analysis revealed that differentially methylated genes that lack an age-related expression change predominantly represent genes involved in carcinogenesis and developmental processes, and expression of most of these genes were silenced in PBMCs. No changes in DNA methylation were found in genes displaying transiently induced changes in gene expression. In conclusion, aging-induced differential methylation often targets developmental genes and occurs mostly without change in gene expression.

**Electronic supplementary material:**

The online version of this article (doi:10.1007/s11357-014-9648-x) contains supplementary material, which is available to authorized users.

## Introduction

The unavoidable and complex process of organismal aging is characterized by a progressive decline in structural and functional features of all organs in the body, resulting in increased morbidity and mortality. Molecular processes underlying these deteriorating effects have been extensively studied in many experimental contexts, but are still only partially understood. Age-induced changes in gene expression have been observed in a broad variety of organisms, and the importance of deregulated gene expression in the process of aging is commonly acknowledged (Lee et al. 1999; Lund et al. 2002; McCarroll et al. 2004; Park and Prolla 2005; Pletcher et al. 2002; Zahn et al. 2007). Epigenetic modifications, including CpG methylation, histone modifications, and regulation by non-coding RNAs, affect gene expression without modifying the DNA sequence (Goldberg et al. 2007). Increasing evidence suggests that these epigenetic modifications might be important mechanisms underlying aging-related changes in gene expression (D'Aquila et al. 2013; Huidobro et al. 2012; Johnson et al. 2012). DNA methylation is up until now the most intensively studied epigenetic mark in aging research. Studies examining age-related changes in DNA methylation started already a long time ago (Berdyshev et al. 1967), but rapid technological advancements during the last 5 years allowing genome-wide analysis of the DNA methylation status have caused a strong acceleration in this research field. In human samples, age-related changes in DNA methylation have been detected in whole-blood (Garagnani et al. 2012; Hannum et al. 2012; Horvath et al. 2012; Rakyan et al. 2010; Teschendorff et al. 2010; Bell et al. 2012) or in purified subsets of blood cells (Heyn et al. 2012; Rakyan et al. 2010), saliva (Bocklandt et al. 2011), brain (Hernandez et al. 2011; Horvath et al. 2012; Numata et al. 2012), or in various other cell and tissue types (Bork et al. 2010; Koch et al. 2011; Koch and Wagner 2011; Teschendorff et al. 2010). These studies have revealed a large number of genes and probe sets displaying either aging-related hyper- or hypomethylation. However, up until now, concomitant changes in gene expression have only marginally been explored on a genome-wide scale.

Most expressed genes show hypomethylation of the promoter region combined with hypermethylation of the gene body (Jones 2012). Changes in the regular DNA methylation pattern, hypermethylation of the promoter regions, and hypomethylation of the gene body have been shown to alter normal expression levels in aging cells and tissues (D’Aquila et al. 2013; Huidobro et al. 2012; Jones 2012). In addition to aging, alterations in gene expression in response to epigenetic modifications have also been reported during normal embryonic development (Cantone and Fisher 2013) or for instance in a disease like cancer (Dawson and Kouzarides 2012), where they cause long-lasting effects. Currently, only a limited number of studies have shown a correlation between transient changes in gene expression and alterations in DNA methylation (Aoi et al. 2011; Barres et al. 2012; Doig et al. 2012; Kangaspeska et al. 2008; Metivier et al. 2008; Pero et al. 2011), and the importance of DNA methylation for transient regulation of gene expression still needs to be established. Expression regulated via the nuclear receptor proliferator-activated receptor alpha (PPARα) can be used as a read-out to analyze the relevance of differential DNA methylation for the regulation of transient changes in gene expression for several reasons. PPARα is a ligand-activated transcription factor involved in the regulation of a variety of processes, ranging from inflammation and immunity to nutrient metabolism and energy homeostasis (Kersten 2010). Long-term effects on gene expression in relation to DNA methylation for PPARα have previously been shown in response to perinatal exposure to a low-protein diet (Lillycrop et al. 2008) and as a consequence of continuous exposure to the peroxisome proliferator WY14,643 (Pogribny et al. 2007). Furthermore, the PPARα target genes PDK4 and FABP4 and the PPARα coactivator PGC-1a have recently been reported to show a DNA-methylation-related change in gene expression (Barres et al. 2012; Kulkarni et al. 2012), so there are strong indications that DNA methylation is involved in PPARα-mediated gene expression. We have previously shown that activation of the PPARα nuclear receptor causes a pronounced change in gene expression in human PBMCs (Bouwens et al. 2008), but whether DNA methylation is involved in this process has not been determined yet.

This study was designed (1) to determine the correlation between age-related DNA methylation and gene expression and (2) to elucidate DNA methylation changes involved in transient changes in gene expression upon WY14,643 treatment. For these purposes, peripheral blood mononuclear cells (PBMCs) were isolated from five young and five old healthy male blood donors. Infinium 450K BeadChips analysis revealed a large number of changes in DNA methylation between young and old subjects, in particularly localized in genes involved in developmental processes. Affymetrix Human Gene 1.1 ST expression arrays showed that expression of most of these genes is silenced and do not display an aging-induced change in gene expression. Moreover, our data show hardly any change in DNA methylation upon WY14,643-treatment and suggest that DNA methylation does not play a causal role in transiently-induced changes in gene expression regulated by this ligand.

## Materials and methods

### PBMC incubation

PBMCs from ten healthy Caucasian male blood donors, aged 30, 31, 34, 35, 43, 52, 62, 64, 65, and 66 years, were isolated directly after arrival of the buffy coat (maximum 8 h after donation) by Ficol-paque Plus density gradient centrifugation (Amersham Biosciences, Roosendaal, The Netherlands). All donors gave full written informed consent. PBMCs were incubated in RPMI1640 medium with 2 mmol/L l-glutamine, 10 % fetal bovine serum, and antibiotics (penicillin and streptomycin) in the presence of 5 % CO_2_ at 37 °C at 1.0 × 10^6^ cells/ml with either WY14,643 (50 μM) or vehicle (DMSO, 0.05 %). After 13 h exposure, the cell suspensions were transferred to 15-ml tubes and centrifuged for 5 min with 1,600 rpm at 4 °C. The two cell pellets per donor were resuspended in ice-cold PBS, and each transferred to separate Eppendorf tubes for RNA and DNA isolation, respectively, and again centrifuged for 5 min at 5,000 rpm at 4 °C. After removing the supernatant, pellets for DNA isolation were snap frozen on dry ice and stored at −80 °C. The pellets for RNA isolation were suspended in 700 μL of buffer RPE with added B-mercaptoethanol according to manufacturer’s instructions (Qiagen) and passed five times through a 23G needle before freezing at −80 °C.

### RNA isolation

Total RNA was isolated using RNeasy Micro Kit from Qiagen according to the manufacturer’s instructions. The RNA was treated with DNAse and purified on columns using the RNeasy microkit (Qiagen, Venlo, The Netherlands). RNA concentration was measured on a NanonDrop ND-1000 ultraviolet–visible spectrophotometer (Isogen, Maarsen, The Netherlands), and RNA integrity was checked on an Agilent 2100 Bioanalyzer (Agilent Technologies, Amsterdam, The Netherlands) with 6000 Nano Chips according to the manufacturer’s instructions. RNA was judged as suitable only if samples showed intact bands of 18S and 28S ribosomal RNA subunits, displayed no chromosomal peaks or RNA degradation products, and had a RNA integrity number (RIN) above 8.0.

### DNA isolation

Genomic DNA was isolated from the isolated PBMC pellets using DNeasy® Blood and Tissue Kit (Qiagen, Venlo, The Netherlands) according to the manufacturer’s instructions. The DNA was treated with RNase and eluted in Qiagen elution buffer AE. DNA purity and quantity were checked spectrophotometricaly (ND-1000, nano-Drop technologies, Wilmington, USA).

### Affymetrix gene expression microarray processing

Total RNA from PBMCs was labeled using an Ambion WT Expression kit (Life Technologies, Bleiswijk, The Netherlands) and hybridized to Affymetrix Human Gene 1.1 ST expression arrays (Affymetrix, Santa Clara, CA, USA). Sample labeling, hybridization to chips, and image scanning were performed according to the manufacturer's instructions on an Affymetrix GeneTitan instrument.

### Affymetrix gene expression microarray data analysis

Array data were analyzed using an in-house, online system (Lin et al. 2011). Shortly, probe sets were redefined according to Dai et al. (2005) using remapped CDF version 15.1 based on the Entrez Gene database. In total, these arrays target 19,682 unique genes. Intensity-based moderated t-statistics (Sartor et al. 2006) was applied to determine the statistical differences between the group of young and old subjects. For the analysis of the WY effect, treated samples were paired with control samples from the same donor, and genes were considered differentially expressed at *p* < 0.01. Functional interpretation of the data was performed through the use of Ingenuity Pathway Analysis (IPA) (Ingenuity® Systems,). Array data have been submitted to the Gene Expression Omnibus, accession number GSE49058.

### Infinium 450K BeaChip analysis

DNAs were prepared in a total volume of 20 μl (1 μg of FF) using a commercial kit EZ DNA Methylation kit (Zymo Research Corp, Orange, CA, USA). A microarray platform (Infinium HumanMethylation450 BeadChips ; Infinium Inc., San Diego, CA, USA) was used, which was processed by the UCL Genomics Core Facility in accordance with the manufacturer’s recommendation. The scanned data and image output files were managed with Genomestudio software (version 1.9.0; Illumina).

### Infinium 450K DNA methylation data analysis

The raw signal intensity values were normalized using the subset-quantile within array normalization (SWAN) method (Maksimovic et al. 2012), as implemented in the minfi R-package. Arrays were then checked for high quality using the control probe information of the Infinium 450K array. Evaluated were (1) the distribution of signal intensity for each of the quality control probes for bisulphite conversion, (2) extension and hybridization in both of the channels, as well as (3) the intensity distribution for 614 negative control probes that are present on the array. Finally, density plots of the methylation beta values of all samples were compared. No outlier arrays were identified, so all arrays were included in the subsequent analyses. Next, probes were filtered that contained a single nucleotide polymorphism (SNP) at or near the target CpG with minor allele frequency equal or larger than 1 % and probes that contained more than 2 SNPs. This resulted in the removal of 18,986 probes. In addition, all non-CpG methylation probes (e.g., CAG, CAH, and CTG) and “rs”(random SNPs) probes were discarded (3,156 probes). Finally, 207 probes were removed that had a detection *p* > 0.05. Taken together, the filtering procedure reduced the total number of 485,577 probes present on the array with 22,349 to 463,228. To identify differentially methylated CpG probes between old and young subjects, the methylated and unmethylated signals were first converted into methylation beta values, where beta was defined as: Beta = Meth / (Meth + Unmeth). Since Beta is a proportion and bounded between 0 and 1, a logit transformation was performed, as recommended for inferential statistical analysis (Du et al. 2010). To avoid dividing with small values or zero, a beta-threshold *ε* of 0.001 was used, so beta values were always in the interval [*ε*; 1 − *ε*]. Finally, differences in *M* values were tested for statistical significance using moderated *t* tests, in which the sample variances were shrunk by computing empirical Bayes posterior means using the limma package (Smyth 2004). Specifically, comparison between age groups were made using only the 10 mock-treated arrays; however, statistical power was increased by including the 10 WY14,643 arrays and treating each subject as random effect using the function duplicate correlation(). The mean beta value of the group of young and old subjects was calculated, and significant differentially methylated probes (*p* < 0.01) displaying at least 5 % methylation difference between the mean value of the young and the mean value of the old subjects (ΔYO > 0.05) were included in the analysis. Statistical significance of WY effects for each subject were inferred by a paired sample comparison. Again, probes were included in the analysis if the methylation difference between the group of treated versus untreated samples was >5 %. 450K data have been submitted to the Gene Expression Omnibus, accession number GSE49064.

## Results

### Aging-induced differential methylation identified by applying Infinium 450K BeadChip analysis

**Fig. 1:**
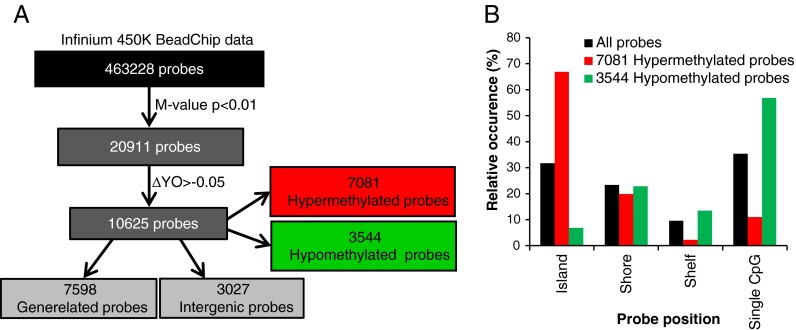
Identification of significantly differentially methylated hyper- and hypomethylated probes. **a** DNA methylation was analyzed in human PCMCs with the Infinium HumanMethylation 450K BeadChips. Of the 463,228 probes included in the analysis 20,917 were significantly regulated (Lima *t* test, *p* < 0.01) between the group of old and young subjects. Over 5 % methylation difference between the mean of the group of the young and old subjects was found for 10,625 of these probes including 7,081 hypermethylated and 3,544 hypomethylated probes. The subset of 10,625 probes contains 7,598 gene-related and 3,027 intergenic probes. **b** Most of the probes displaying hypermethylation in the group of old subjects are located in CpG islands, while hypomethylated probes were predominantly found at single CpG sites

To evaluate genome-wide effects of aging and WY14,643-treatment on DNA methylation and gene expression, PBMCs were isolated from 10 healthy male Caucasian volunteers ranging in age from 30 to 66 years. The mean age of the group of the five young subjects was 34.6 and of the five old subjects 61.8 years (age of the individual subjects in the two groups is presented in supplemental Table 1). As shown in Fig. 1a, 20,911 of the total number of 463,228 probes included in the analysis were found to be significantly (*p* < 0.01) differentially methylated in mock-treated PBMCs (see “Materials and methods”). Of these significant differentially methylated probes, 10,625 showed a methylation difference of at least 5 % between the mean value of the group of young and old subjects (ΔYO > 5 %) including 7,081 hyper- and 3,544 hypomethylated probes. The results presented in Fig. 1b show that the majority of all probes measured on the 450K Infinium BeadChips are either located in a CpG island or at a single CpG site. In line with previous studies (Christensen et al. 2009; Hannum et al. 2012; Heyn et al. 2012), we found that the aging-related hypermethylated probes were predominantly present in the CpG islands, while the majority of the hypomethylated probes were found in less CpG dense regions (shelves and single CpG dinucleotides). Of the 10,625 probes containing a methylation difference of at least 5 %, 3,027 are intergenic probes while 7,598 are linked to one or more genes, in total associated with 4,370 unique genes.

**Table 1:** Aging-related differentially expressed genes with four or more differentially methylated probes

Number of differentially methylated probes per gene	4	5	6	7	8	9	10	11
Gene symbols	AHRR^a^	BAI1^a^	ADAM12	EFCAB1	FOXI2	FBXO39	*TBX15*	DOCK1^a^
	BCL2L2	ERBB4^a^	IGF2BP1^a^	*NETO1*	*INS-IGF2* ^a^			
	C12orf34	LYPD1	*HS3ST2* ^a^		*TNF*			
	CCK^a^	OSR2	MYO1D^a^					
	*CDH2*	SH3BP2	SLC9A3^a^					
	CDK2AP1	TMEM132C						
	COL23A1^a^							
	CYFIP1^a^							
	FAT1							
	FOXA2							
	FZD10							
	HAS1							
	HIC1							
	ISLR2							
	*MANEAL*							
	NCOR2							
	*PAX5*							
	PROM1							
	RANBP17^a^							
	*SP140*							
	TRIL							
	UGGT2							
	*WDR8* ^a^							

Symbols in italics indicate up-regulated expression; symbols in normal font indicate down-regulated expression

^a^Contains hypo- and hypermethylated probes

To ensure the results presented in this study are not caused by an aging-induced change in cell type, we checked the cell mixture composition in our samples by applying the previously reported method of Houseman et al. (2012) on the 450K data of our PBMC samples. We implemented this algorithm using the estimateCellCounts function in the minfi R-package. The results obtained clearly show that the purification of the blood samples completely removed, as intended, the granulocyte fraction of the leucocytes and that there were no significant differences in blood cell composition in the PBMCs between the group of young and old subjects (see supplemental Fig. 1).

### Identification of epigenetically controlled aging-induced changes in gene expression in PBMCs

Genome-wide gene expression analysis revealed that, of the above described 7,598 differentially methylated gene-related probes, 640 were not represented on the Affymetrix Human Gene 1.1 ST expression array. By evaluating the 407 genes associated to these 640 probes in more detail, we found that, apart from a number of pseudo- and uncharacterized genes, this subset of probes contained antisense RNAs, long intergenic nonprotein coding RNAs and micro-RNAs (data not shown), suggesting the involvement of epigenetic mechanisms other than DNA methylation in the aging process.

**Fig. 2:**
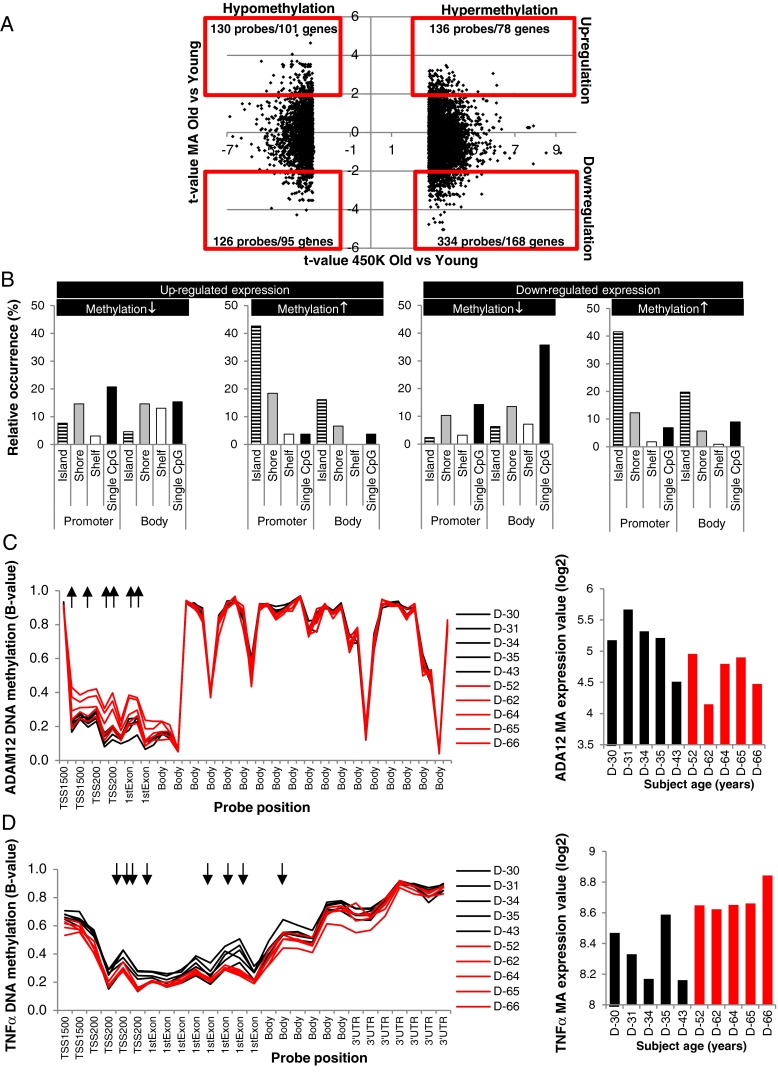
Differential expression of genes associated to differentially methylated gene-related probes. **a** *t* values of the 10,625 differentially methylated probes are plotted against the MA *t* values. The most pronounced changes in gene expression are identified by applying −2 > MA *t* value > 2 as a cut-off and presented in the *redlined squares*. **b** Relative occurrence of the probe location is presented for all hyper- and hypomethylated probes linked to the differentially expressed genes. **c** Beta-values of all probes associated to *ADAM12* present on the 450K BeadChips, six of them show significant hypermethylation (↑) in the promoter region of the gene of old subjects, accompanied by down-regulated gene expression. **d** Methylation profile of the 450 K TNF-α probes reveal significant hypomethylation (↓) of eight probes in old individuals in the promoter region as well as in the gene body together with up-regulated gene expression

*T* values of the remaining 6958 probes were plotted against the microarray (MA) *t* values of the related genes (Fig. 2a), and the most significant differentially expressed genes were identified using −2 > *t* value > 2 as a cut-off. As shown in Fig. 2a, 470 probes were hypermethylated, of which 334 were concomitant with down-regulation of gene expression in 168 genes and 136 linked to up-regulation of 78 genes. Of the 256 hypomethylated probes, 130 accompanied 101 up-regulated genes, while 126 probes were linked with 95 down-regulated genes. We next analyzed the localization and CpG density (island, shore, shelf, or single CpG) of the hyper- and hypomethylated probes linked to differentially expressed genes. The results revealed that hypermethylated probes of both up- and down-regulated genes are mostly located in CpG-dense promoter regions [probes located in TSS1500, TSS200, 5′ untranslated region (UTR) and first exon regions] and to a lesser extent in CpG-dense regions of the gene bodies (Fig. 2b). The hypomethylated probes did not reveal a clear methylation profile but showed a more random distribution over the promoter and body region with a slight enrichment for CpG poor regions. Since hardly any differentially methylated probes were found in the 3′UTR regions, these data were not included in Fig. 2b.

Of all 398 differentially regulated genes presented in Fig. 2a (and listed in supplemental Table 2S), 170 contained multiple differentially methylated probes and for a number of genes both hypo- as well as hypermethylated probes were detected. Table 1 displays all genes containing at least four differentially methylated probes. In Fig. 2c and d, two examples of differentially expressed genes containing multiple differentially methylated probes are presented. Disintegrin and metalloproteinase domain-containing protein 12 (ADAM12) is an example of a gene displaying age-related down-regulated gene expression. As shown in Fig. 2c in the group of old subjects, significant hypermethylation in the ADAM12 promoter was observed. Different aging-induced differential methylation was observed for tumor necrosis factor α (TNF-α). As shown in Fig. 2d, for TNF-α aging-induced up-regulated gene expression combined with significant hypomethylation of eight probes present in the promoter as well as in the gene body was found.

Taken together, a subset of genes was identified displaying differential expression as well as differential methylation.

### A large subset of genes display age-related differential methylation without change in gene expression

**Fig. 3:**
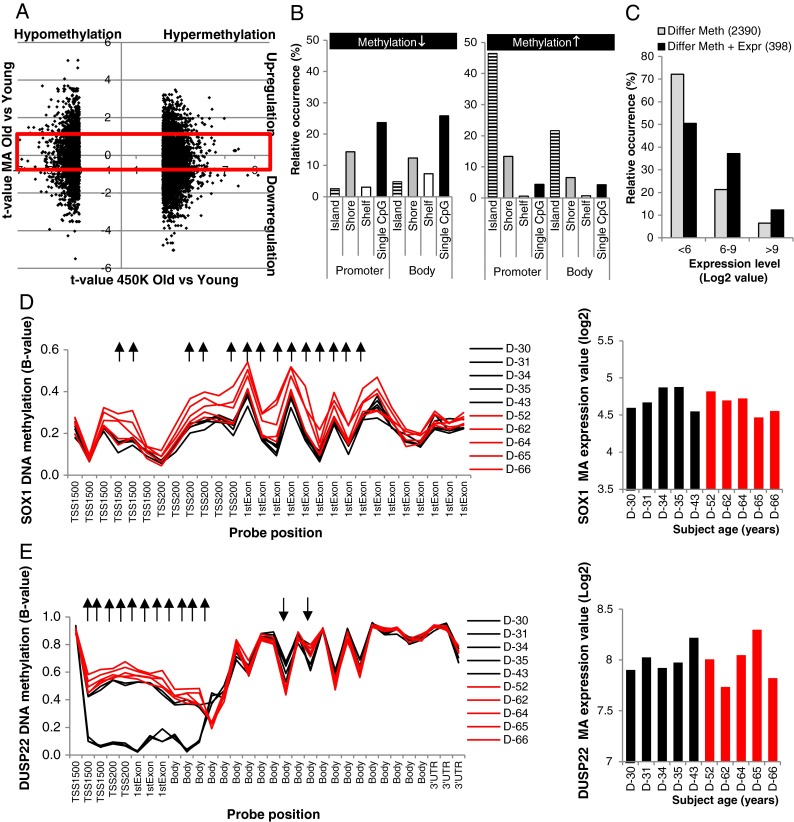
Genes associated to a large number of differentially methylated probes do not display a change in gene expression. **a** 2,390 genes present in the *redlined square* are associated to 4,554 differentially methylated probes and do not display a change in gene expression (−1 < MA *t* value < 1). **b** Relative occurrence of the probe location is presented for all hypo- and hypermethylated differentially methylated probes. **c** Basal expression of the genes lacking an age-induced change in gene expression is lower than that of genes displaying an age-related change in gene expression. **d** DNA methylation profile of the *SOX1* gene displays age-related hypermethylation, but the expression of this gene is not changed in old compared to young subjects. **e** Strong hypomethylation was observed in the promoter region of three of the five young subjects, but no change in expression of the *DUSP22* gene was detectable

Next, we focused our analysis on the aging-induced differentially methylated probes lacking a concomitant change in gene expression. Figure 3a shows that 4,554 differentially methylated probes (listed in supplemental Table 3S) occur without changing the expression status of the 2,390 genes they are associated with (using −1 < *t* value < 1 as a cut-off for the MA data). Again, many genes are represented by multiple differentially methylated probes, and the number of differentially methylated probes per gene reaches much higher numbers (Table 2) than observed for the genes displaying age-related differential expression (Table 1). We analyzed the probe localization and CpG density of all hyper- and hypomethylated probes. As shown in Fig. 3b, hypomethylation of the genes lacking a change in gene expression occurs predominantly at single CpGs in the promoter region as well as in the gene body. The hypermethylated probes were predominantly located in the CpG dense regions of the promoter and, to a lesser extent, in the gene bodies. This DNA methylation profile is highly similar to the hypermethylated probes encoding for aging-induced differentially expressed genes presented in Fig. 2b. By evaluating the basal expression levels of the two subsets of genes, we found that more than 70 % of the genes displaying aging-induced changes in DNA methylation without a concomitant change in gene expression have extremely low expression levels (MA log2 < 6) (see Fig. 3c). Higher basal expression was observed for the genes displaying an age-related change in gene expression. Promoter hypermethylation has previously been shown to cause down-regulated gene expression (Jones 2012), so, if expression of a particular gene is already extremely low, hypermethylation of the promoter regions might occur without altering gene expression. An example of such a gene is SRY-related HMG-box-1 (SOX1), a gene that has also previously been reported to show aging-related changes in DNA methylation (Bell et al. 2012; Horvath et al. 2012; Teschendorff et al. 2010). All probes present on the 450K BeadChip linked to SOX1 are located in the promoter region of this gene and 14 of them reveal significant aging-induced hypermethylation (see Fig. 3d), which does not result in a further decrease of the low expression values present at young age. Zic family member 1 (ZIC1) is the gene containing the highest number (20) of age-related differentially methylated probes and is located on chromosome 14 in close proximity to ZIC4 for which 17 differentially methylated probes were found. Both genes display age-related hypermethylation at all differentially methylated probes located in the promoter regions as well as in the gene bodies (see supplemental Fig. 2S), but the already extreme low expression levels in young individuals are not further reduced in the older individuals (data not shown). However, the lack of change in gene expression cannot be explained by low basal expression levels in all cases. In three of the five young subjects, dual specificity phosphatase 22 (DUSP22) shows markedly decreased methylation of the promoter region combined with increased methylation in the gene body, but these methylation changes did not alter the moderate expression levels of this gene (Fig. 3e). Since age-related differential methylation of DUSP22 has not been reported in previous studies and our study contained a low sample size, the variation in DNA methylation might not necessarily be caused by aging but might reflect interindividual variation.

**Table 2:** Aging-related differentially methylated genes with eight or more differentially methylated probes but lacking a change in gene expression

Number of differentially methylated probes per gene	8	9	10	11	12	13	14	17	18	19	20
Gene symbols	CA10	DPP6	GPR158	KANK4	PCDHGA12	DUSP22	PCDHA12	PCDHA7	TNXB	PCDHGA1	ZIC1
	DIO3	EYA4	PLEC1	PCDHA2		FOXG1	SIM1	ZIC4			
	GABRA5	FLRT2		PCDHGA9			SOX1				
	GRIA2	MAGI2									
	KHDRBS2	NRG1									
	SFRP2	PAX3									
	SHISA6	PITX2									
	SOX11	SPTBN4									
	ZNF454										

Infinium 450K BeadChip data methylation data were validated by analyzing the methylation status of several CpGs by pyrosequencing, while Affymetrix expression data were validated by applying quantitative PCR (Q-PCR) analysis. The obtained results are presented in supplemental Fig. 3S + 4S.

In summary, differential methylation of multiple probes representing the same gene is found for many genes that do not show an age-related change in gene expression. Silenced expression of many of these genes may be (partially) responsible for the absence of an expression change. Although these prominent epigenetic changes seem to be without phenotypic consequences, they may be useful as biomarkers of aging.

### Differential methylation detectable in developmental genes and genes involved general cellular processes

**Fig. 4:**
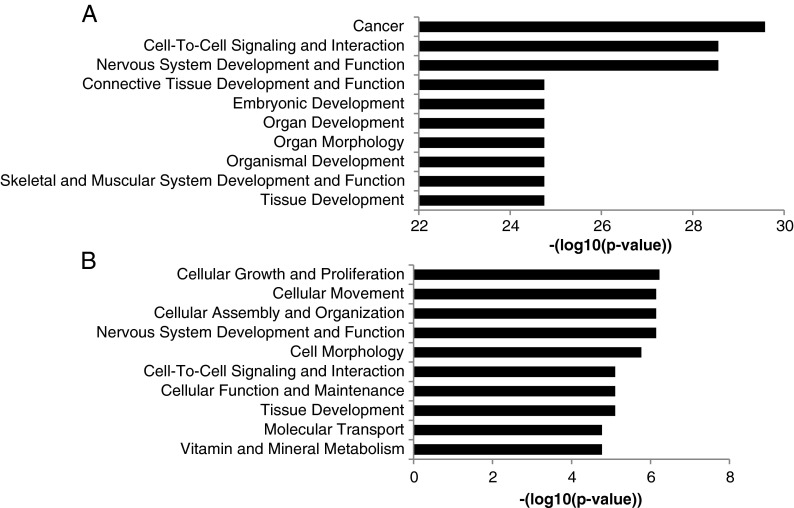
Functional analysis of genes associated to differentially methylated probes. **a** Differentially methylated probes linked to genes lacking a change in gene expression are predominantly involved in carcinogenesis and developmental processes. **b** Genes displaying an aging-induced change in gene expression containing differentially methylated CpGs function in various basal cellular processes

To evaluate the functions of the differential methylated genes, IPA was applied. Interestingly, most of the 2,390 genes described above that display one or more differentially methylated probes but lack an age-related change in gene expression were found to be involved in cancer and in various developmental processes (see Fig. 4a). Different functional categories were observed when the genes displaying differential methylation as well as differential expression were evaluated. As shown in Fig. 4b, these genes were found to be involved in different basal cellular processes like cellular growth and proliferation, cellular movement, cell/tissue morphology, and metabolic functions.

### Previously published potential biomarkers of aging are not differentially expressed in PBMCs of old subjects

During the last few years, several research groups have identified aging-related changes in DNA methylation by analyzing either Infinium 27K or 450K BeadChips in whole-blood (Bell et al. 2012; Florath et al. 2013; Garagnani et al. 2012; Hannum et al. 2012; Teschendorff et al. 2010; Xu and Taylor 2014) or in purified blood cell samples (Heyn et al. 2012; Rakyan et al. 2010). These studies reporting aging-related changes in DNA methylation in blood samples applied different selection criteria and the number of reported differentially methylated probes varied from 9 (Garagnani et al. 2012) to 5,988 (Heyn et al. 2012). In total, 7,477 different differentially methylated probes have been reported in these eight studies (see supplemental Table 4S), of which just a limited set of 529 probes have been reported by more than one group. From this subset of 529 probes, we selected the genes reported with the highest frequency in the above mentioned 8 studies, which are represented by at least 3 age-related differentially methylated probes. As shown in Table 3, this list includes ELOVL fatty acid elongase 2 (ELOVL2), four and a half LIM domains 2 (FHL2), proenkephalin (PENK), Krüppel-like factor 14 (KLF14), somatostatin (SST), and glycine receptor, alpha 1 (GLRA1) that have previously been put forward as epigenetic biomarkers of aging (Garagnani et al. 2012; Hannum et al. 2012; Heyn et al. 2012). Of all of these previously reported epigenetic biomarkers of aging at least one, but in most cases multiple, probes showed significant (*p* < 0.01 and ΔYO > 5 %) differential methylation in our PBMC data set. Intriguingly, none of these genes displayed aging-related differential expression in PBMCs.

**Table 3:**
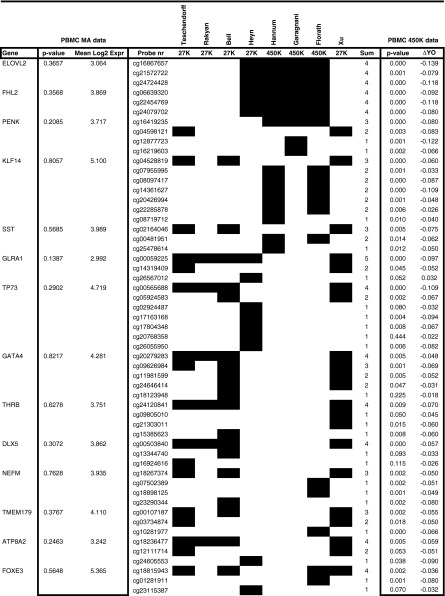
Genes displaying multiple differentially methylated probes in various previously reported studies

In summary, substantial variation was found in aging-induced changes in DNA methylation by applying 27K and 450K analysis in white blood cells as reported in recently published studies. However, a number of markers are detected in multiple studies and might be useful biomarkers of aging, but they lack an age-related change in gene expression in PBMCs.

### WY treatment of PBMCs did result in a pronounced change in gene expression without detectable change in DNA methylation

**Fig. 5:**
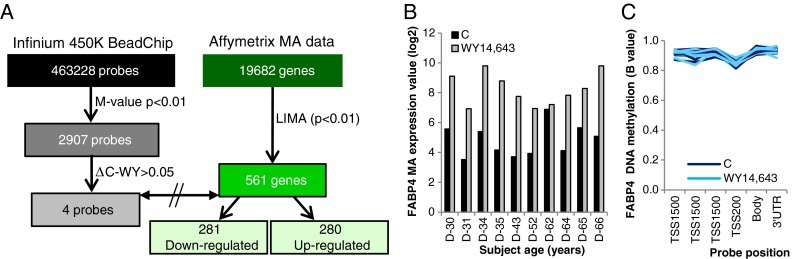
WY14,643 treatment of PBMCs induced a strong response in microarray gene expression but caused only a minor effect on DNA methylation. **a** In response to WY14,643-treatment 2907 probes displayed significant (*p* < 0.01) differential methylation 4 of which containing over 5 % change between control and Wy-treated samples. These probes were not associated to the 561 genes displaying significant (*p* < 0.01) differential expression. **b** Strong induction of FAPB4 expression upon WY treatment. **c** DNA methylation of the six probes representing the highest PPARα-response gene *FABP4* on the 450K array did not show a change in DNA methylation upon WY14,643 treatment

To evaluate whether transiently induced changes in gene expression are correlated to changes in DNA methylation, PBMCs of all donors were cultured for 13 h in the presence of the PPARα ligand WY14,643 (50 μM) or vehicle. As seen in Fig. 5a, 2,907 of the probes showed significant differential methylation in response to WY14,643 treatment. Of four of these probes, the difference in methylation between control and WY-treated samples was over 5 % (ΔC-WY > 0.05). Microarray expression analysis revealed that 561 genes were significantly (*p* < 0.01) differentially expressed upon WY treatment, including 281 down-regulated and 280 up-regulated genes. The four differentially methylated probes (ΔC-WY > 0.05) were not associated to the differentially expressed genes.

It should be noted that the CpG probes examined with the 450K BeadChips often represent only a minority of the total number of CpGs present in a particular gene. The strongest WY-induced gene in our data set, fatty acid binding protein 4 (FABP4) (see Fig. 5b), contains 42 CpG sites (from TSS1500 through to 3′UTR), but only six of them are measured on the 450K BeadChip (Fig. 5c). We examined the methylation status of two additional CpG sites present in the promoter of the FAPB4 gene that are not present on 450K BeadChips and found no change in DNA methylation upon treatment with WY14,643 (see supplemental Fig. 3S) in line with the results obtained of the 450K BeadChip analysis.

Taken together, the results obtained show that WY14,643 treatment caused only marginal changes in DNA methylation, indicating that WY14,643-induced changes in gene expression are not caused by alterations in DNA methylation.

## Discussion

In this study, Infinium 450K BeadChip analysis was applied to identify age-related genome-wide changes in DNA methylation in human PBMCs. The obtained results revealed significant differential methylation of 10,625 probes displaying a methylation change of at least 5 % between the group of young and old subjects. Functional analysis of the genes associated with these differentially methylated probes revealed strong enrichment of genes involved in cancer and in an extensive number of developmental gene clusters. Aging-related differential methylation of genes involved in developmental processes has also been observed in previous studies. Bork et al. (2010) found enrichment in the differential methylation of a specific subset of developmental genes, the HOX genes, in mesenchymal stromal cells in response to aging. Furthermore, aging-related hypermethylation of polycomb target genes have been reported by Maegawa et al. (2010) in the intestine, by Teschendorff et al. (2010) in different cell types and by Beerman colleagues in hematopoietic stem cells (Beerman et al. 2013). Aging-induced differential methylation of developmental genes has recently been reported by Rakyan et al. (2010) in purified white blood cells, and our data support this. Intriguingly, our results show that a wider variety of developmental genes are differentially methylated during aging than previously described and also indicate that genes involved in carcinogenesis are differentially methylated. It is interesting to note that genes in which expression is tightly regulated by epigenetic mechanisms during embryonic organ, tissue, and cellular development display the most pronounced loss of their regular DNA methylation pattern during the process of aging. It can be speculated that enzymes responsible for the tight regulation of the DNA methylation (Jurkowska et al. 2011) or demethylation (Bhutani et al. 2011; Wu and Zhang 2010) gradually lose their capacity to keep the accurate hyper- and hypomethylation status of the CpGs in these genes intact during the process of aging causing erosion of the epigenome. Interestingly, our data reveal that these changes mostly occur without changing the expression levels of the developmental genes and that expression of most of these genes is silenced in PBMCs. Emerging data suggest that deregulation of genes playing an essential role in early development leads to various pathologies including carcinogenesis (Dormoy et al. 2012) in adulthood. The fact that aging-related changes in DNA methylation do not result in gene expression changes might prevent disease development and reflect “healthy aging.” It can be speculated that when, in addition to the changes in DNA methylation, gene mutations, histone modifications, or deregulation of miRNAs occur, expression levels of these genes might change resulting in a diseased phenotype. Alternatively, in tissues where these genes are expressed, changes in DNA methylation might result in concomitant altered expression levels in contrast to the situation observed in PBMCs.

A relatively small subset of genes displayed an age-induced change in DNA methylation as well as in gene expression. By analyzing the probe location and CpG content of the DNA region, we found highly similar features for the subsets of genes with and without change in gene expression so this could not explain the discrepancy between the two gene sets. In addition, we compared the basal expression levels of the two subsets of genes and found lower basal expression in the differentially methylated genes lacking a concomitant change in gene expression compared to the subset of genes that exhibited changes in both aspects. It can be speculated that a change in DNA methylation might not result in altered expression levels when relevant transcription factors are absent; however, low basal expression levels cannot fully explain why differential DNA methylation in some of the genes does not result in a change in gene expression. For instance, DUSP22 showed intermediate basal expression levels but pronounced changes in DNA methylation did not seem to affect gene expression (see Fig. 3e). On the other hand, for ADAM12, differential expression was observed in old subjects, despite the fact that this gene showed extremely low basal expression levels (see Fig. 2c). For some of the differentially methylated genes, the detected changes in DNA methylation might be located in parts of the gene not involved in the regulation of gene expression and thereby do not result in a change of the expression levels of the related gene. Furthermore, it should be noted that a change in gene body methylation may not alter gene expression as it is measured on a microarray but it may be regulating gene expression via alternative splicing programs (Li-Byarlay et al. 2013).

Functional analysis of the differentially methylated genes displaying altered gene expression revealed that a variety of functional categories representing general cellular functions like cell growth, movement, signaling, and development were found to be differentially expressed with high significance. This observation implies that DNA methylation might be responsible for the functional loss of common cellular functions in the process of aging and thereby play a causal role in the development of the aging phenotype. Unexpectedly, we rarely observed differential expression of immune system-related genes in the PBMCs. The most pronounced immune-related effect was found for TNF-α showing enhanced expression combined with decreased methylation in older individuals. Increased TNF-α levels contribute to inflammaging, and our results are in line with the age-related loss of TNF-α promoter methylation recently reported by Gowers et al. (2011).

Since our data set is based upon a limited number of samples, we concentrated our analysis on the more robust effects and omitted minor changes in order to reduce the risk of false positive results. It has previously been reported that, due to impaired functioning of the bone marrow as well as the thymus, skewing toward myelopoiesis can alter the balance between monocyte and lymphocyte cell fractions between old and young individuals (Chinn et al. 2012). Cell mixture distribution analysis of our samples revealed that there was no significant difference in the cell type populations present in the PBMCs isolated from the healthy young and old subjects included in this study.

Previous genome-wide DNA methylation studies applying Infinium 27K or 450K BeadChip analysis in blood samples have identified a substantial number of potential epigenetic biomarkers of aging. In the future, these biomarkers might be used to determine a person’s biological age, predict the risk of age-related diseases or be useful in forensic research. By comparing the results of these previous studies with the age-related changes in DNA methylation in our PBMCs, different interesting observations were made. Firstly, our data reveal that the majority of the CpGs displaying age-related differential methylation are only reported in one study. The discrepancy between the reported results might be explained by different reasons. First of all, most of the probes present on the 450K BeadChip are not present on the 27K BeadChip, and some of the probes present on the 27K BeadChips are not on the 450K BeadChips. Secondly, most of the publications used different statistical methods and a different *p* value cut-off for the list of probes presented in the publication. Standardization of microarray preprocessing and statistical analysis methods for large DNA methylation datasets, along with agreed norms for cut-off values, is likely to help build consensus among different research groups. Interestingly, genes that have previously been identified as potential epigenetic biomarkers of aging like ELOVL2, FHL2, PENK (Garagnani et al. 2012), KLF14, SST (Hannum et al. 2012), and GLRA1 (Heyn et al. 2012) have been detected in different studies and are represented by multiple differentially methylated probes. Differential methylation of all of these genes was confirmed in our PBMC data set, but none of them displayed age-related differential expression. In addition, Weidner and colleagues recently identified three novel CpGs that can be used to analyze aging in blood (Weidner et al. 2014). These three CpGs (cg02228185, cg25809905, and cg17861230) showed significant age-related differential methylation in our PBMC dataset, but expression of the genes they encode for (PDE4C, ASPA, and ITGA2B) is extremely low in PBMCs and does alter in aging subjects (see supplemental Table 4S). This result indicates that previously reported epigenetic biomarkers of aging might be useful to analyze epigenetic aging but may not predict phenotypic changes. Furthermore, our comparison revealed additional candidates with an age-related methylation change on multiple probes in our PBMC data set (i.e., TP73, GATA4, FOXE3, THRB, and TMEM179) that have been identified in multiple studies but have not been put forward as potential epigenetic biomarker of aging. Again, none of these genes showed an age-related change in gene expression in PBMCs. As indicated above, the sample size of our study is limited, and therefore, further validation of these results is required in studies with a larger sample size.

Since blood cells can easily be obtained compared to other tissues or organs (i.e., liver, intestine, or brain samples) whole-blood or purified blood cells are often used for biomedical research as an indicator for biological processes occurring at other places in the body. Further studies are required to establish the extent at which blood cells represent the DNA methylation profiles of other cell types particularly for age-related changes in DNA methylation. For genes like TP73, TMEM179, and GLRA1, age-related changes in DNA methylation have been previously reported in saliva, brain, and other tissues (Bocklandt et al. 2011; Horvath et al. 2012; Koch and Wagner 2011; Numata et al. 2012) pointing towards a general phenomenon. However, owing to the fact that tissue-specific differentially methylated regions (tDMRs) have been identified (Rakyan et al. 2008; Fernandez et al. 2012), it seems likely that part of the aging-related changes relate to a tissue-dependent phenomenon. The relation between age-related changes in DNA methylation and gene expression might be linked to this observation. Although we did not observe altered expression of SST in PBMCs, an age-related decline in expression has previously been reported in the brain (Hayashi et al. 1997; Lu et al. 2004), and this decline has been linked to Alzheimer's disease (Saito et al. 2005). Genes like ELOVL2, FLH2, and PENK are not expressed in blood cells but are found in heart, smooth muscle, testes, and specific areas of the brain. It would be of interest to examine whether these genes display age-related changes in expression in addition to differential methylation in these tissues.

In addition to the long-term effects of aging, we analyzed the correlation between a transient change in gene expression and DNA methylation. In line with our previous study (Bouwens et al. 2008), WY14,643-induced differential gene expression was observed in PBMCs. Genome-wide DNA methylation analysis with the 450K BeadChip revealed almost no changes in DNA methylation in the same samples, suggesting that expression regulation of the 561 differentially expressed genes is not regulated by DNA methylation. The number of studies reporting a correlation between altered DNA methylation and a transient change in gene expression in adult differentiated cells is extremely limited. It can be speculated that enzymes involved in DNA methylation (Jurkowska et al. 2011) and demethylation (Bhutani et al. 2011; Wu and Zhang 2010) are not able to modify the genome fast enough to induce a transient effect or that additional cell proliferation is required (Hervouet et al. 2012). Furthermore, the correlation between alterations in DNA methylation and concomitant changes in gene expression has predominantly been reported under conditions reflecting long-term effects such as embryonic development (Cantone and Fisher 2013), (stem)cell differentiation (Armstrong 2012; Cantone and Fisher 2013; Cedar and Bergman 2011; Hu and Rosenfeld 2012) in utero nutrition (Heijmans et al. 2008; Lillycrop et al. 2008; van Straten et al. 2010), cancer (Dawson and Kouzarides 2012; Dawson et al. 2009; Hammoud et al. 2013; Portela and Esteller 2010), neurological development and disease (Jakovcevski and Akbarian 2012), etc. It can be hypothesized that, in mature adult cells, other factors like enhancer binding proteins, co-activators and co-repressors, histone modifications, and/or non-coding RNAs dominate transient regulation of gene expression instead of DNA methylation.

In conclusion, genome-wide DNA methylation analysis revealed differential methylation of a wide range of developmental genes in old subjects. Expression of most of these genes was silenced in PBMCs, and differential DNA methylation occurred without a concomitant change in gene expression.

## Electronic supplementary material

Below is the link to the electronic supplementary material.

ESM 1 (PPT 434 kb)

ESM 2 (XLS 7166 kb)
