# Supplementary material for: Genome-wide age-related changes in DNA methylation and gene expression in human PBMCs
Source: Age (Dordr). 2014 May 2;36(3):9648. doi: 10.1007/s11357-014-9648-x (PMC4082572; doi:10.1007/s11357-014-9648-x)
Supplement: Supplementary file 1 — (PPT 434 kb) [file 11357_2014_9648_MOESM1_ESM.ppt]

## Slide 1
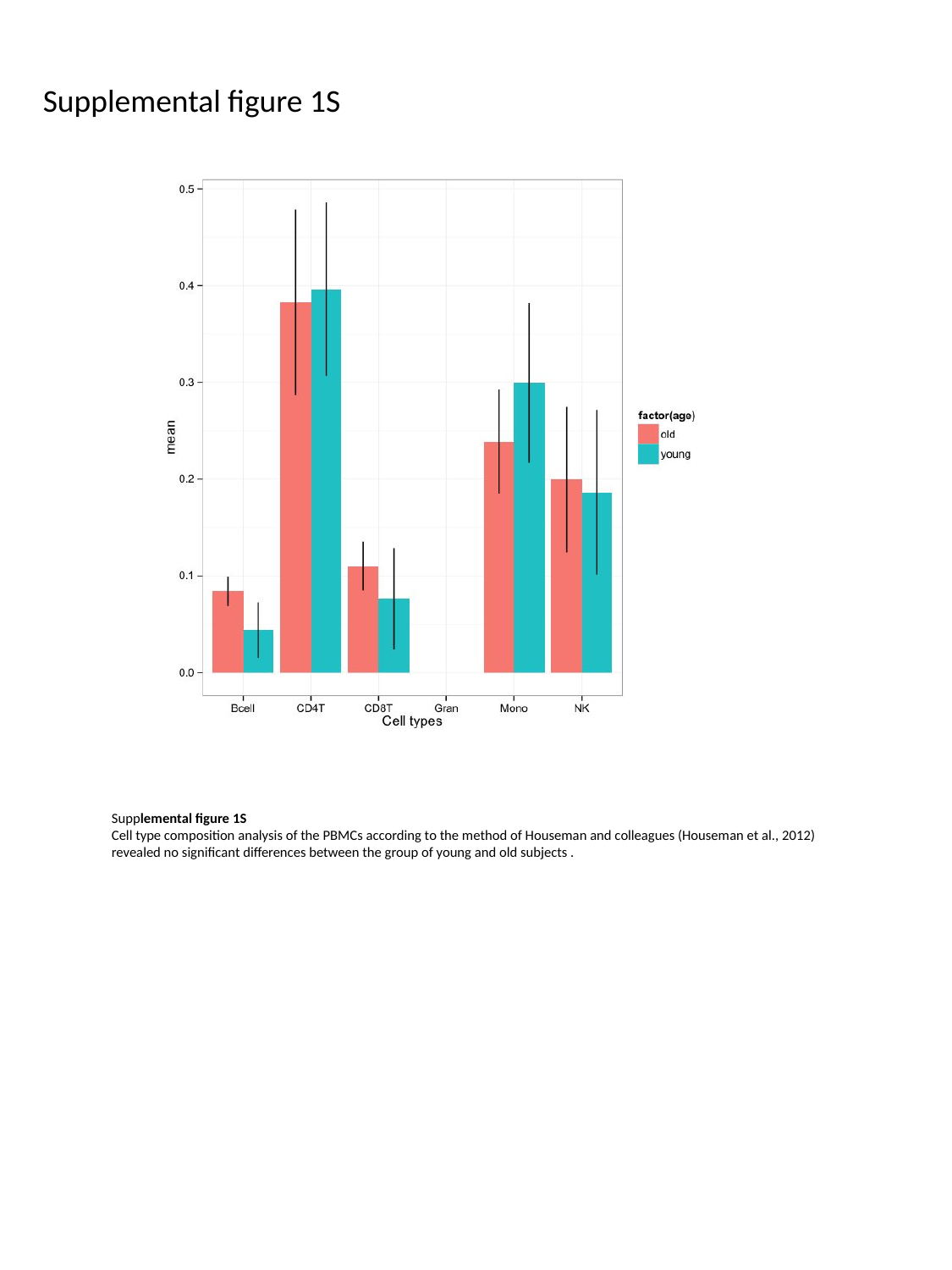

Supplemental figure 1S
Supplemental figure 1S
Cell type composition analysis of the PBMCs according to the method of Houseman and colleagues (Houseman et al., 2012) revealed no significant differences between the group of young and old subjects .

## Slide 2
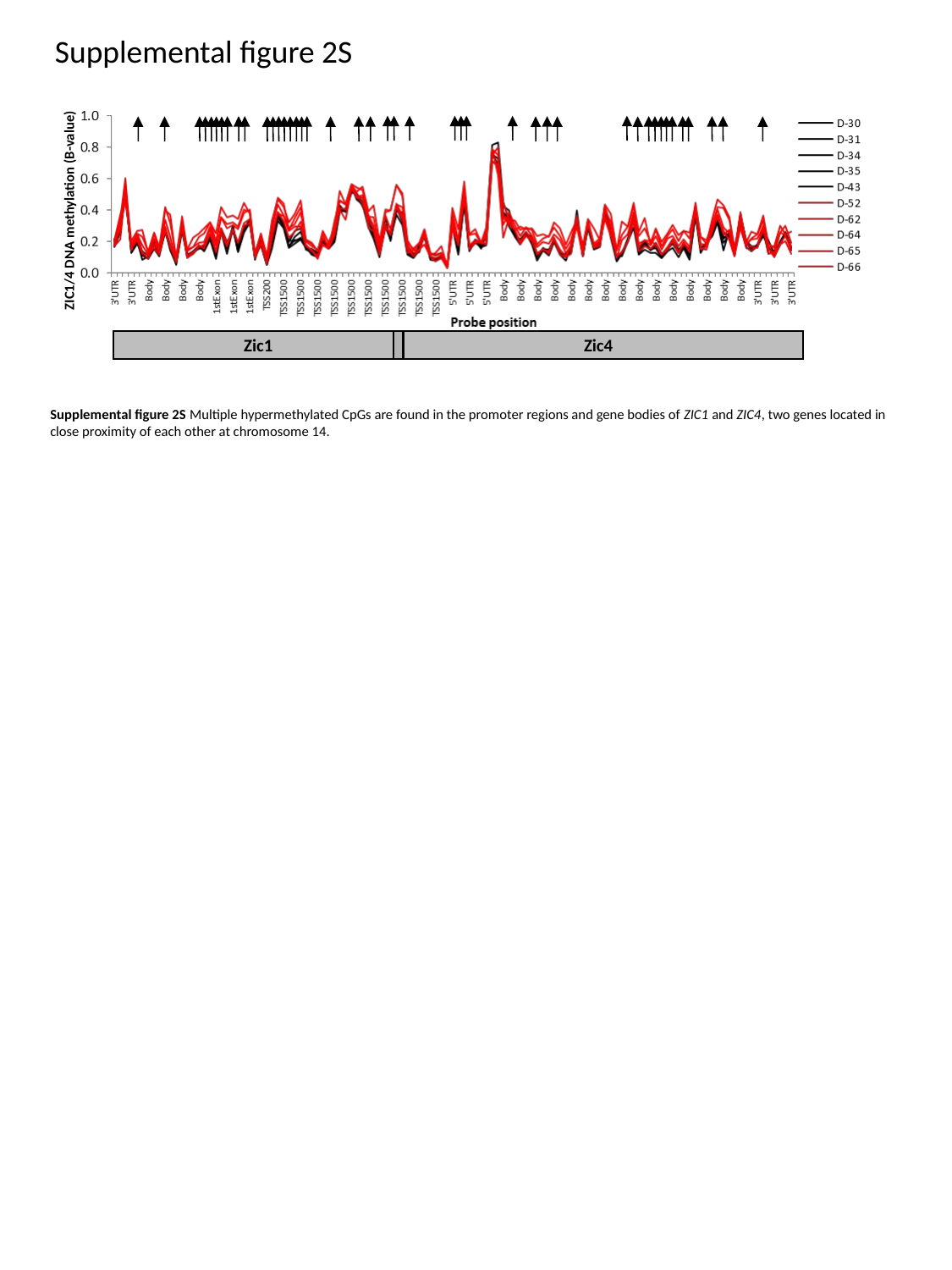

Supplemental figure 2S
ZIC1/4 DNA methylation (B-value)
Zic1
Zic4
Supplemental figure 2S Multiple hypermethylated CpGs are found in the promoter regions and gene bodies of ZIC1 and ZIC4, two genes located in close proximity of each other at chromosome 14.

## Slide 3
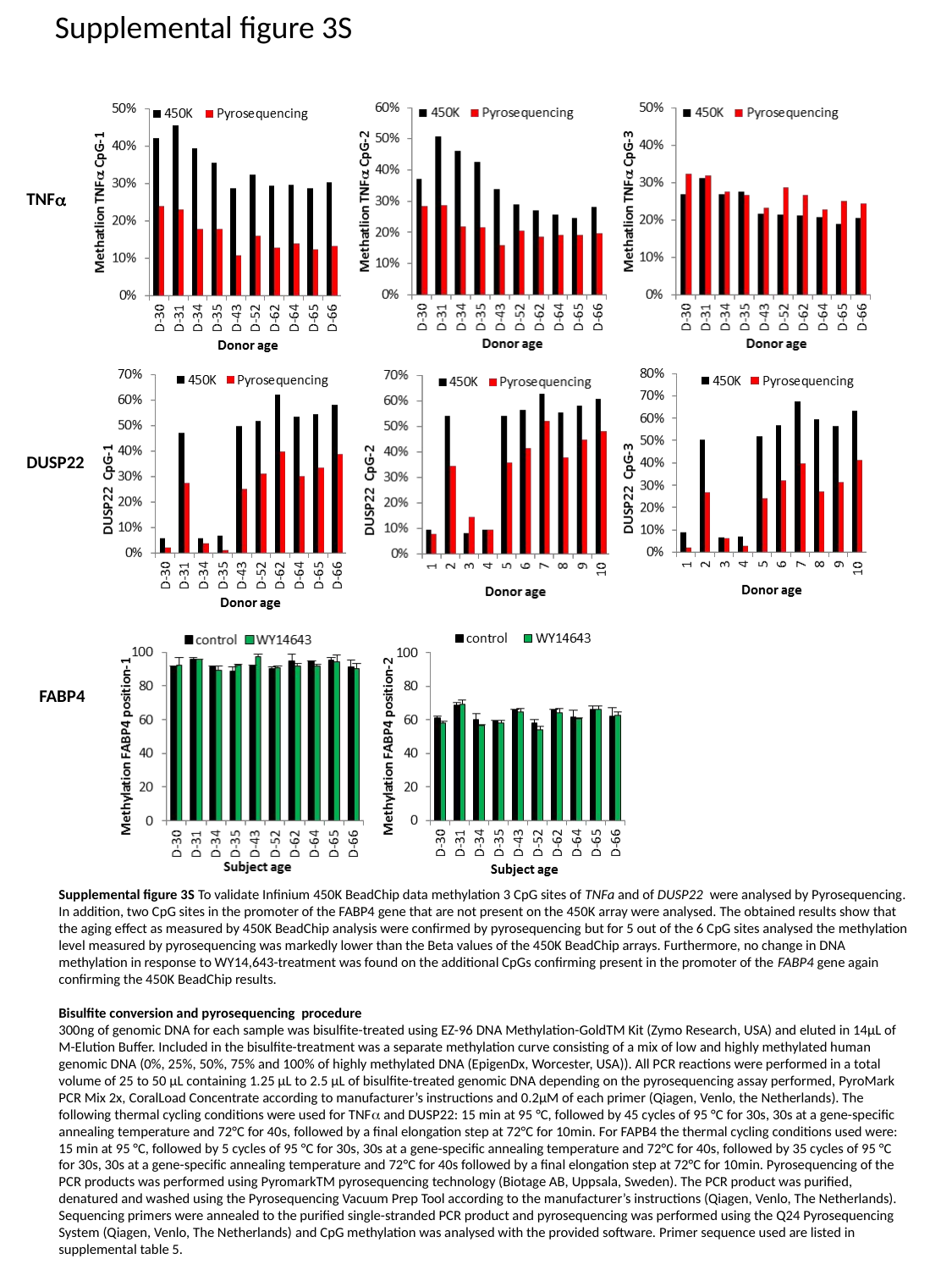

Supplemental figure 3S
TNFα
DUSP22
FABP4
Supplemental figure 3S To validate Infinium 450K BeadChip data methylation 3 CpG sites of TNFa and of DUSP22 were analysed by Pyrosequencing. In addition, two CpG sites in the promoter of the FABP4 gene that are not present on the 450K array were analysed. The obtained results show that the aging effect as measured by 450K BeadChip analysis were confirmed by pyrosequencing but for 5 out of the 6 CpG sites analysed the methylation level measured by pyrosequencing was markedly lower than the Beta values of the 450K BeadChip arrays. Furthermore, no change in DNA methylation in response to WY14,643-treatment was found on the additional CpGs confirming present in the promoter of the FABP4 gene again confirming the 450K BeadChip results.
Bisulfite conversion and pyrosequencing procedure
300ng of genomic DNA for each sample was bisulfite-treated using EZ-96 DNA Methylation-GoldTM Kit (Zymo Research, USA) and eluted in 14μL of M-Elution Buffer. Included in the bisulfite-treatment was a separate methylation curve consisting of a mix of low and highly methylated human genomic DNA (0%, 25%, 50%, 75% and 100% of highly methylated DNA (EpigenDx, Worcester, USA)). All PCR reactions were performed in a total volume of 25 to 50 μL containing 1.25 μL to 2.5 μL of bisulfite-treated genomic DNA depending on the pyrosequencing assay performed, PyroMark PCR Mix 2x, CoralLoad Concentrate according to manufacturer’s instructions and 0.2μM of each primer (Qiagen, Venlo, the Netherlands). The following thermal cycling conditions were used for TNFα and DUSP22: 15 min at 95 °C, followed by 45 cycles of 95 °C for 30s, 30s at a gene-specific annealing temperature and 72°C for 40s, followed by a final elongation step at 72°C for 10min. For FAPB4 the thermal cycling conditions used were: 15 min at 95 °C, followed by 5 cycles of 95 °C for 30s, 30s at a gene-specific annealing temperature and 72°C for 40s, followed by 35 cycles of 95 °C for 30s, 30s at a gene-specific annealing temperature and 72°C for 40s followed by a final elongation step at 72°C for 10min. Pyrosequencing of the PCR products was performed using PyromarkTM pyrosequencing technology (Biotage AB, Uppsala, Sweden). The PCR product was purified, denatured and washed using the Pyrosequencing Vacuum Prep Tool according to the manufacturer’s instructions (Qiagen, Venlo, The Netherlands). Sequencing primers were annealed to the purified single-stranded PCR product and pyrosequencing was performed using the Q24 Pyrosequencing System (Qiagen, Venlo, The Netherlands) and CpG methylation was analysed with the provided software. Primer sequence used are listed in supplemental table 5.

## Slide 4
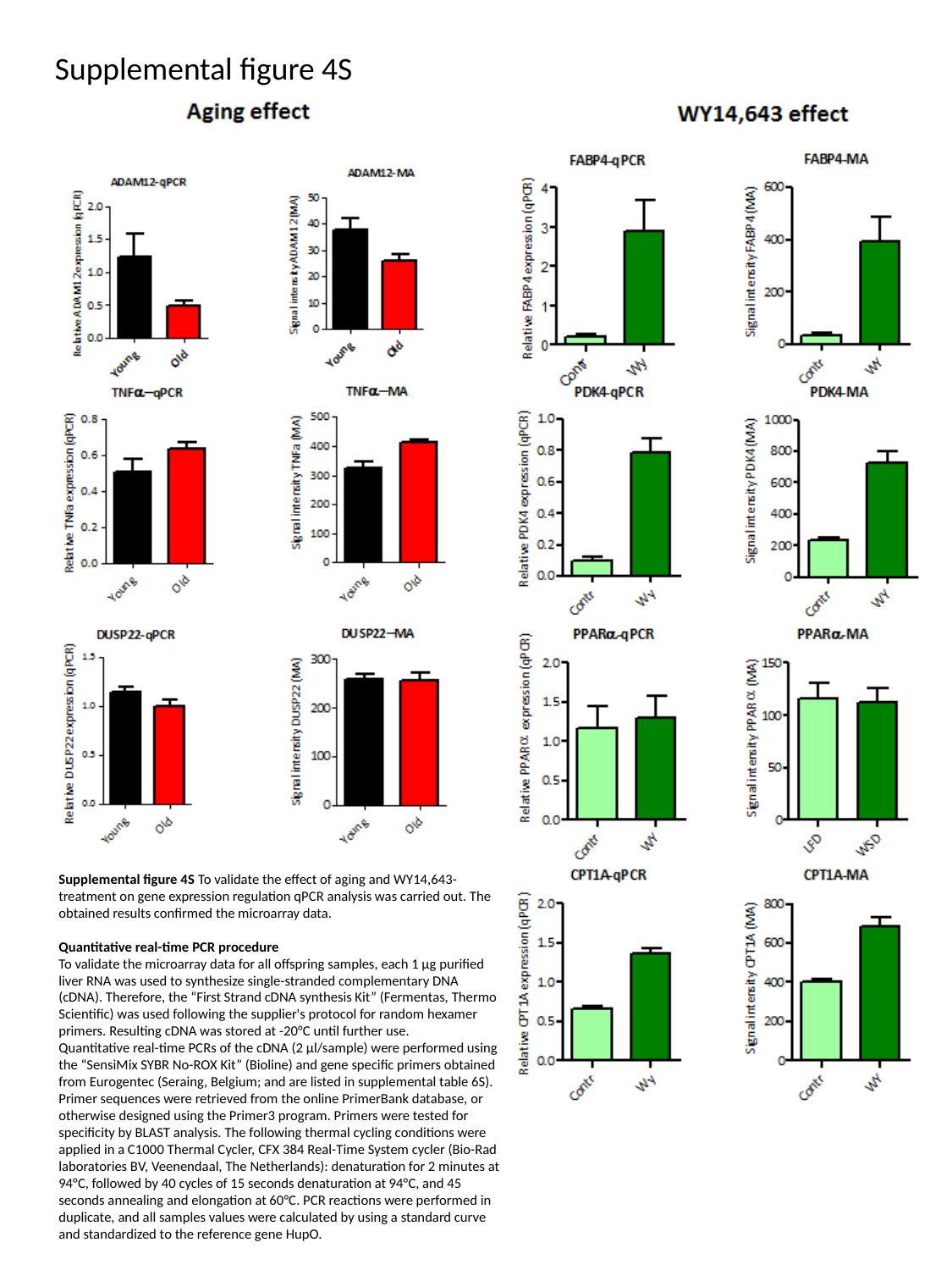

Supplemental figure 4S
Supplemental figure 4S To validate the effect of aging and WY14,643-treatment on gene expression regulation qPCR analysis was carried out. The obtained results confirmed the microarray data.
Quantitative real-time PCR procedure
To validate the microarray data for all offspring samples, each 1 µg purified liver RNA was used to synthesize single-stranded complementary DNA (cDNA). Therefore, the “First Strand cDNA synthesis Kit” (Fermentas, Thermo Scientific) was used following the supplier's protocol for random hexamer primers. Resulting cDNA was stored at -20°C until further use.
Quantitative real-time PCRs of the cDNA (2 µl/sample) were performed using the “SensiMix SYBR No-ROX Kit” (Bioline) and gene specific primers obtained from Eurogentec (Seraing, Belgium; and are listed in supplemental table 6S). Primer sequences were retrieved from the online PrimerBank database, or otherwise designed using the Primer3 program. Primers were tested for specificity by BLAST analysis. The following thermal cycling conditions were applied in a C1000 Thermal Cycler, CFX 384 Real-Time System cycler (Bio-Rad laboratories BV, Veenendaal, The Netherlands): denaturation for 2 minutes at 94°C, followed by 40 cycles of 15 seconds denaturation at 94°C, and 45 seconds annealing and elongation at 60°C. PCR reactions were performed in duplicate, and all samples values were calculated by using a standard curve and standardized to the reference gene HupO.
